# Streptococcus mitis Peritonitis in a Peritoneal Dialysis Patient: A Case Report Highlighting the Importance of Dental Hygiene

**DOI:** 10.7759/cureus.64693

**Published:** 2024-07-16

**Authors:** Patnarin Kanjanabuch, Athiphat Banjongjit, Sirirat Purisinsith, Piyaporn Towannang, Talerngsak Kanjanabuch

**Affiliations:** 1 Department of Oral Medicine, Faculty of Dentistry, Chulalongkorn University, Bangkok, THA; 2 Nephrology Unit, Department of Medicine, Vichaiyut Hospital, Bangkok, THA; 3 Health Department, Bangkok Metropolitan Administration, Bangkok, THA; 4 Continuous Ambulatory Peritoneal Dialysis (CAPD) Excellent Center, King Chulalongkorn Memorial Hospital, Bangkok, THA; 5 Division of Nephrology, Department of Medicine, Faculty of Medicine, Chulalongkorn University, Bangkok, THA; 6 Center of Excellence in Kidney Metabolic Disorders, Faculty of Medicine, Chulalongkorn University, Bangkok, THA

**Keywords:** viridans streptococci, peritoneal dialysis, peritonitis, streptococcus mitis, streptococcus oralis

## Abstract

Viridans-group streptococci, including the *Streptococcus mitis/oralis* subgroup, can cause peritoneal dialysis (PD)-related peritonitis. The link between dental pathology and PD-related peritonitis remains to be fully elucidated. We report a case of an 83-year-old man undergoing nocturnal intermittent PD due to kidney failure from diabetic nephropathy who developed *S. mitis* peritonitis and septicemia traced back to a periodontal abscess. Despite having no prior history of peritonitis and maintaining good nutritional status, the patient presented with generalized abdominal pain and a low-grade fever. The initial treatment included intraperitoneal antibiotics. Root cause analysis identified multiple periodontitis and dental abscesses as the primary source of infection, confirmed by DNA sequencing of cultures from the abscesses and blood, which matched *S. mitis*. This case highlights the critical role of oral flora in causing invasive diseases in immunocompromised individuals, including PD patients, and illustrates how dental infections can lead to PD-related peritonitis through hematogenous spread. Our case also stresses the importance of meticulous dental care and regular dental examinations to prevent such infections in PD patients.

## Introduction

Dental problems can lead to Viridans-group streptococcal septicemia. Occasionally, researchers have reported the *Streptococcus mitis/oralis* subgroup as a cause of peritoneal dialysis (PD)-related peritonitis [1-5]. However, the connection between PD-related peritonitis caused by the *Streptococcus mitis*/*oralis* subgroup and dental issues remains unclear. We describe a case where a patient undergoing PD developed *S. mitis* peritonitis and septicemia linked to a periodontal abscess.

## Case presentation

An 83-year-old man with a history of kidney failure due to diabetic nephropathy was receiving assisted nocturnal intermittent PD. His treatment included 1.5% dextrose 2L exchanges five times daily, lasting 12 hours, with ultrafiltration ranging from 500 to 1000 mL/day. He had been under this regimen for one year, managed by a paid caregiver. His medical history also included hypertension, hypothyroidism, asthma, and moderate aortic regurgitation, along with well-controlled diabetes (his glycated hemoglobin was below 7%). The patient had lost all residual kidney function and had no previous instances of peritonitis. His nutritional status was excellent, with a subjective global assessment [6] of grade A and a serum albumin level of 3.6 g/dL. A dental examination before commencing PD revealed no concerns, but he did not maintain regular dental check-ups afterward.

On presentation, he reported generalized abdominal pain and a low-grade fever lasting one day. Initial analysis of his PD effluent (PDE) revealed a leukocyte count of 10,505 cells/µL, with 96% neutrophils. Based on these findings, he was diagnosed with PD-associated peritonitis and septicemia. Treatment started immediately with piperacillin/tazobactam 3.375 g intravenous (IV) every six hours on day 1, followed by 2.25 g IV every eight hours on day 2. Then, switching to intraperitoneal cefazolin and ceftazidime, both at 1 g, which were administered on day 3 since the fever subsided and the clinical condition improved. Subsequent leukocyte counts in the PDE decreased to 143 cells/µL by day 3, dropped further to 10 cells/µL by day 5, and have consistently remained below 10 cells/µL thereafter. A culture of the day-1 PDE identified the *Streptococcus mitis/oralis* subgroup by day 3, showing susceptibility to all commercial antibiotics, including penicillin, cefazolin, and piperacillin/tazobactam. This prompted a treatment adjustment following the 2022 International Society for PD (ISPD) Peritonitis Guidelines [7]. Cefazolin was continued for 14 days (days 3-14). Peripheral blood cultures also identified the *Streptococcus mitis/oralis* subgroup.

To further identify the bacteria, we conducted a standard bacterial polymerase chain reaction and DNA sequencing of the 16S ribosomal RNA (rRNA) with a universal bacterial primer (800 bp) [8]. This analysis showed a 99.6% and 99.8% match, respectively, in the PDE and blood cultures to *S. mitis* (accession numbers MT538298.1 and MT512123.1), according to the BLASTN (Basic Local Alignment Search Tool for Nucleotides) program results.

**Figure 1:**
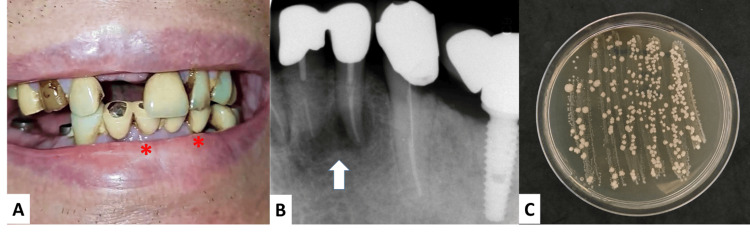
(A) An intraoral photograph showing poor oral hygiene, with visible calculus and stains on the generalized teeth, missing teeth, and a fixed prosthesis. (B) An intraoral radiograph displays apical radiolucency around the roots of the lower left incisors (indicated by a white arrow) and radiolucency beneath the crown of the lower left canine, indicative of dental abscesses. (C) Cultures on Tryptic Soy Agar from the dental abscesses showing off-white bacterial colonies.

We conducted a root cause analysis following the Plan-Do-Study-Act (PDSA) cycle to identify the primary source of the infection, guided by the identified pathogen. This involved surveying the patient/caregiver with self-reported Oral Health Impact Profile (OHIP) questionnaires [9] and performing gastroendoscopy and colonoscopy. On day 3, an examination of the oral cavity revealed multiple instances of periodontitis (Figure 1A).

An intraoral radiograph showed apical radiolucency around the roots of the lower left incisors and beneath the crown of the lower left canine, indicating dental abscesses (Figure 1B). We extracted three teeth. Cultures from the abscesses grew several organisms, including *Enterococcus faecalis*, *Proteus mirabilis*, *Klebsiella pneumoniae*, and *Viridans streptococcus*, which were later identified as *S. mitis* (Figure 1C). The caregiver confirmed adherence to aseptic protocols, including handwashing, mask usage, and PD exchange procedures. We also conducted upper and lower gastrointestinal endoscopies to search for other sources of primary pathology, which only revealed multiple small colonic polyps. Biopsies of these polyps did not show any malignant cells. Additionally, repeated transthoracic echocardiograms found no evidence of valvular vegetation or abscess. As of this writing, the patient has been free from recurrent peritonitis for two years.

## Discussion

In this report, we describe a patient with PD-related peritonitis infected by *S. mitis*, with the same pathogen detected in both dental abscesses and the bloodstream. This confirms that dental pathology was the primary source of the infection, with secondary hematogenous peritoneal seeding rather than the usual touch contamination leading to peritonitis and secondary bacteremia.

The *S. mitis*/*oralis* subgroup, part of the Viridans group of streptococci and characterized as α-hemolytic Streptococcus, includes species like *S. oralis* and *S. mitis*. These bacteria are typically part of the commensal flora in the oral cavity, gastrointestinal tract, and female genital tract. Still, they can cause invasive diseases in individuals with compromised immune systems, such as infective endocarditis, endophthalmitis, and meningitis [10]. Sequencing the 16S rRNA gene has been crucial in identifying Viridans-group streptococci, leading to the discovery of novel species from clinical samples [11]. Initially, we identified the organism as the *S. mitis*/*oralis* subgroup. Subsequent DNA barcoding accurately identified the species as *S. mitis* in all samples.

Peritonitis may arise from several routes: intraluminal (from touch contamination), periluminal (related to the catheter), transmural (from the intestinal tract), ascending (from the female genital tract), and hematogenous [12]. Different bacteria are associated with each route, for example, Coagulase-negative Staphylococcus from intraluminal contamination, *Staphylococcus aureus* from catheter exit-site infections, and α-hemolytic streptococci from bloodstream infections following dental procedures [13]. PD-related peritonitis due to oral pathogens has been reported previously [1-5], but these cases lacked peripheral blood culture growth, leaving the connection unclear. Our case illustrates a direct pathogenesis from a *Streptococcus mitis* dental infection to peritonitis via hematogenous spread.

The 2022 ISPD guidelines recommend a two-week course of suitable antibiotics for treating streptococcal peritonitis [7]. Most Viridans-group streptococci infections (68%) respond to penicillin or first-generation cephalosporins. However, penicillin resistance among these bacteria has decreased from 77% between 2005 and 2009 to 57% between 2010 and 2014 [14]. In our case, the pathogen showed susceptibility to penicillin/cefazolin with a minimum inhibitory concentration of less than 0.023 µg/mL. Koruk et al. [5] reported a strain of the *S. mitis*/*oralis* subgroup resistant to penicillin but susceptible to cefazolin. Most reported cases achieved a medical cure, except for one [3] that recurred, suspected to be related to playing the saxophone. No cases documented bacteremia, making it difficult to definitively pinpoint the source of infection (Table 1) [1-5,15].

**Table 1:** Review literature of Streptococcus mitis/oralis PD-related peritonitis PD, peritoneal dialysis; IP, intraperitoneal; IV, intravenous; N/A, not available. Remark: None of the reported species have confirmed the inoculation with DNA barcoding.

First author, year	Age	Sex	Organism	Antibiotics and duration	Outcome	Suspected source
Mert [2], 2022	32	Female	S. mitis	Ceftazidime and cefazolin IP 14 days	Medical cure	Transluminal (diarrhea) and poor oral hygiene
Mert [2], 2022	72	Female	S. mitis	Ceftazidime and cefazolin IP 14 days	Medical cure	Transluminal (diarrhea) and poor oral hygiene
Mizuno [1], 2011	54	Female	S. mitis	Vancomycin IV	Medical cure	N/A
Amirou [3], 2012	77	Male	S. mitis/oralis	Amoxicillin + clavulanic acid 15 days	Recurrent	Suspected saliva disseminated by saxophone
Kotani [4], 2021	77	Male	S. oralis	Cefazolin and ceftazidime IV 22 days	Medical cure	Unknown
Koruk [5], 2005	40	Female	S. oralis	Cefazolin IP 10 days	Medical cure	Unknown
Mihara [13], 2023	60	Male	S. oralis	Meropenem and Vancomycin IP followed by Cefazolin IV 3 weeks	Medical cure	Suspected dental issue

## Conclusions

We have reported a case of PD-related peritonitis caused by *S. mitis*, resulting from hematogenous spread from a dental infection. This case highlights the critical role of regular dental check-ups for PD patients before and after starting PD. The case also underscores the importance of investigating oral pathology when peritonitis occurs due to Viridans-group streptococci.
